# Antibiotic-Resistant *Shigella sonnei* Bacteremia in an Immunocompetent Postgastric Bypass Patient Without Typical Risk Factors

**DOI:** 10.1155/crdi/9910105

**Published:** 2025-03-27

**Authors:** Alejandra Gutierrez, Shovendra Gautam

**Affiliations:** Department of Internal Medicine, Texas Christian University Anne Burnett Marion School of Medicine, Fort Worth, Texas, USA

## Abstract

*Shigella* bacteremia, though rare, is a serious condition that highlights the importance of recognizing potential complications in patients with *Shigella* infections. In this paper, we present a case of *Shigella* bacteremia in a 53-year-old female s/p a Roux-en-Y gastric bypass revision four months prior, presenting with acute onset of nausea, vomiting, and persistent, explosive, watery diarrhea which started one day after she consumed pork chops and ground beef. Initial laboratory tests indicated leukocytosis and hypokalemia. Human immunodeficiency virus (HIV), rapid plasma regain (RPR), and Hepatitis C were nonreactive. A computed tomography (CT) scan of the abdomen and pelvis showed no intestinal wall thickening or any other acute abnormalities. Blood cultures identified a *Shigella sonnei* infection that was resistant to multiple antibiotics. She was treated with intravenous meropenem. *Shigella* bacteremia is a rare complication of *Shigella* infection and requires thorough investigation to identify potential underlying factors. Antibiotic susceptibilities should also be assessed, given the increasing resistance of *Shigella* strains to previously effective treatments.

## 1. Introduction

*Shigella* species are Gram-negative bacilli that are nonmotile, nonspore-forming, and facultatively anaerobic, known for causing diarrheal illnesses by invading the epithelial lining of the colon but rarely causing bloodstream infections. The *Shigella* genus is categorized into four serogroups: *Shigella dysenteriae*, *Shigella flexneri*, *Shigella boydii*, and *S. sonnei*, each containing multiple serotypes [1]. Transmission typically occurs through the consumption of contaminated food and water, especially in areas with poor sanitation and limited access to clean water. Commonly implicated foods include cold salads, vegetables, lettuce, meat, and dairy products [2]. In addition, *Shigella* can spread through fecal-oral transmission, posing a higher risk to men who have sex with men, particularly those with compromised immune systems [3]. Current international literature reports, a significant number of patients with *Shigella* bacteremia are infected with HIV [4]. In the United States, case series have reported *Shigella* bacteremia occurring in children under 1 year of age and in adults suffering from malnutrition, HIV infection, and other immunocompromising conditions such as diabetes mellitus and malignancies [5].

## 2. Case Presentation

A 53-year-old female with a past medical history significant for congestive heart failure, hypertension, hyperlipidemia, and obesity s/p Roux-en-Y gastric bypass presented to the Emergency Department with a chief complaint of nausea, vomiting, and diarrhea. The symptoms began suddenly with uncontrollable vomiting and diarrhea. She reported that the vomiting has subsided, but the diarrhea persists, occurring multiple times daily. She described the diarrhea as explosive, watery, nonbloody, and uncontrollable, often preceded by a burning sensation in her anal region. She has experienced bowel incontinence multiple times. She had a recent Roux-en-Y gastric bypass revision 4 months before this incident. The day before symptom onset, the patient attended a church dinner at a local soul food restaurant where she consumed pork chops and ground beef. Notably, her dog, who also ate the ground beef, experienced similar gastrointestinal symptoms. The patient denied any recent sick contacts or knowledge of any other church members experiencing similar symptoms, antibiotic use, fevers, chills, chest pain, shortness of breath, abdominal pain, melena, hematochezia, saddle anesthesia, or dysuria.

On examination, the patient was alert, awake, and obese. She was afebrile. The abdomen was soft, nontender, and nondistended, with hyperactive bowel sounds. Notable lower extremity edema was present, worse on the left. There were no signs of nuchal rigidity, focal neurological deficits, or episodes of inattentiveness. All other findings were within normal limits. Given her clinical presentation and recent dietary changes, differential diagnoses considered were infectious gastroenteritis, food poisoning, or complications related to her recent gastric bypass revision.

The patient was admitted from the ED with an initial WBC count of 11.3, which peaked at 19 during the admission before gradually decreasing to 15.5, lactic acid level of 0.6, serum creatinine of 1.5, and potassium of 2.8, with no acute EKG changes. Potassium was replaced as needed. A CT abdomen/pelvis without contrast was obtained to evaluate for a source of infection and it showed a prominent gallbladder, postgastric bypass changes, and a normal appendix without any intestinal wall thickening. X-rays revealed no acute fractures. Urinalysis showed 0–5 WBCs, 2+ leukocyte esterase, positive nitrites, and many bacteria. *Clostridium difficile* antigen test was negative. HIV, RPR, and Hepatitis C were nonreactive. She was empirically started on ceftriaxone and metronidazole. Antibiotic susceptibility testing was performed using standard microdilution methods in accordance with Clinical and Laboratory Standards Institute (CLSI) guidelines. Blood cultures were positive for *Shigella sonnei*, resistant to ampicillin and sulfamethoxazole-trimethoprim, with intermediate resistance to ciprofloxacin, but susceptible to levofloxacin. After one day of ceftriaxone and metronidazole, she was switched to meropenem 1 g every 8 h. By the third day, daily stool output steadily trended downward and the patient began feeling less nauseous. The patient's diarrhea frequency steadily decreased from seven to eight episodes per day on admission to two to three episodes by day 3 of hospitalization. In addition, while the patient was afebrile on presentation, serial temperature monitoring remained within normal limits throughout hospitalization, further supporting clinical improvement. Repeat blood cultures were negative. It was recommended that the patient continue Meropenem 1 g every 8 h for a total of 10 days.

## 3. Discussion

*Shigella* species are commonly found in temperate and tropical regions. In high-income countries*, S. sonnei* is the predominant cause of shigellosis, whereas *S. flexneri* is more common in low- and middle-income countries. Infections by *S. boydii* and *S. dysenteriae* are less frequent globally; *S. boydii* is mainly seen in the Indian subcontinent, while *S. dysenteriae* is mostly found in sub-Saharan Africa and South Asia [2]. Globally, *Shigella* is responsible for an estimated 80–165 million cases of illness and 600,000 deaths each year, predominantly affecting children. Of these cases, around 20–119 million illnesses and 6900–30,000 deaths are attributed to foodborne transmission. Foodborne outbreaks have been reported among travelers, with tourists contracting the illness from contaminated food in hotels, on airplanes, and on cruise ships. In rare instances, *Shigella* bacteria can penetrate the damaged intestinal lining and lead to a bloodstream infection [2, 6].

Bacteremia is more frequently observed in young children and adults over 65 years compared with older children and younger adults [7]. Another study identified young age and malnutrition as the two primary host factors associated with an increased risk of *Shigella* bacteremia in patients [8]. A study on systemic shigellosis in South Africa found that most cases (95%) were diagnosed via blood cultures. HIV prevalence among cases with *Shigella* bacteremia was 67%, highest among patients aged 5–54 years and more common in females (79%) than males (52%). HIV-infected individuals were 4.1 times more likely to die from shigellosis compared to uninfected individuals (37% vs. 13%) [4].

In contrast, our patient does not fit this typical demographic profile. She is 53 years old, neither very young nor elderly, and does not have HIV or other common immunocompromising conditions. Her presentation of *Shigella* bacteremia is unusual given the absence of these typical risk factors. Instead, her risk may be linked to recent gastrointestinal surgery (Roux-en-Y gastric bypass revision 4 months prior) and potentially other factors like diet changes and exposure to contaminated food, which may have contributed to her development of the infection. Understanding nontraditional risk factors for *Shigella* bacteremia is crucial for broadening the scope of at-risk individuals and improving clinical practice. For example, recent gastrointestinal surgeries, such as our patient's Roux-en-Y gastric bypass revision, can significantly alter gut flora and immune responses, making patients more susceptible to infections. The surgical modification of the digestive system can lead to malabsorption and changes in gut microbiota, potentially weakening the gut's natural defenses against pathogens like *Shigella*. In addition, dietary factors play a significant role. Our patient experienced a sudden onset of symptoms following a significant dietary change, consuming potentially contaminated food, such as pork chops and ground beef, which her dog also ate and subsequently showed similar symptoms. While *Shigella* species are well-documented causes of diarrheal disease, their ability to cause invasive bloodstream infections has been predominantly reported in immunocompromised populations, particularly individuals with HIV, malnutrition, or chronic illnesses such as diabetes. There was one case reported in the literature about *Shigella sonnei* bacteremia in an elderly diabetic patient who presented with acute febrile gastroenteritis. Blood cultures were positive for *S. sonnei*, while stool cultures were negative. The patient was treated with appropriate antibiotics and recovered without complications. Unlike our immunocompetent patient with a history of gastric bypass surgery, this case involved an elderly individual with diabetes, a known risk factor for *Shigella* bacteremia [9].

A key aspect of this case is the patient's recent Roux-en-Y gastric bypass revision, which may have altered gut immunity and increased susceptibility to bacterial translocation. *Shigella sonnei* invades the bloodstream by translocating through the intestinal epithelium, often via microfold (M) cells, and inducing epithelial disruption through inflammation, facilitating bacterial entry into the circulatory system [10]. The role of gastrointestinal surgery in facilitating bacteremia remains underexplored, but it is plausible that structural and functional changes following bariatric surgery, including alterations in the gut microbiome and transient mucosal permeability, could predispose patients to systemic infections. Preventive strategies should focus on strict food hygiene, ensuring proper cooking of meats, and minimizing exposure to contaminated food or water, particularly in individuals with recent gastrointestinal surgeries. In addition, early recognition of symptoms and prompt medical evaluation in postsurgical patients presenting with severe diarrhea may help prevent progression to bacteremia [6].

A study titled *Shigella sonnei Bacteremia in Adults: A Report of Five Cases and Review of the Literature* described five adult patients with *Shigella* bacteremia and reviewed 22 additional cases. The majority of the patients had underlying predisposing conditions or were older than 65 years, and their clinical presentations commonly included acute febrile gastroenteritis. Notably, in some cases, *Shigella* was isolated from blood cultures even when stool cultures were negative [7]. In contrast, the absence of underlying predisposing conditions in our patient differentiates this case from most of the patients in the reviewed study, who had significant comorbidities, reinforcing the importance of considering alternative risk factors, such as recent gastrointestinal surgery, in the development of *Shigella* infection.

According to the Centers for Disease Control and Prevention (CDC), *Shigella* species have become resistant to numerous antibiotics over time. The CDC classifies XDR (extensively drug-resistant) *Shigella* as strains resistant to azithromycin, ciprofloxacin, ceftriaxone, trimethoprim-sulfamethoxazole, and ampicillin. Due to the increasing prevalence of antimicrobial resistance, it is vital to conduct susceptibility testing for *Shigella*. Specifically, ciprofloxacin susceptibility testing should include drug dilutions of 0.12 mcg/mL or lower [11].

In our case, this patient was treated with meropenem. Throughout the course of treatment, the patient's WBC count initially increased from 11.3 on admission to a peak of 19 before gradually decreasing to 15.5 as clinical symptoms improved. Fosfomycin and meropenem are potential treatment options for XDR *Shigella* infections, although comprehensive clinical data are not yet available. The CDC has not identified resistance to meropenem among *Shigella* isolates, and fosfomycin resistance remains uncommon. However, there has been insufficient study of both fosfomycin and meropenem in the context of shigellosis. While limited data exist for the use of fosfomycin in treating infectious diarrhea, most of this research has been performed outside the U.S. and applies to pathogens other than *Shigella* [12].

An epidemiological study in the UK described an outbreak of XDR *S. sonnei*, involving 72 cases of multidrug-resistant or XDR isolates. Phenotypic testing revealed that all isolates were susceptible to ertapenem, meropenem, temocillin, and fosfomycin. The study recommended oral pivmecillinam and fosfomycin for cases with prolonged symptoms or as a step-down therapy after intravenous (IV) antibiotic treatment. For hospitalized patients with suspected or confirmed complicated or severe infections, meropenem or ertapenem were suggested as IV treatment options [13].

Fosfomycin has shown activity against various Gram-negative pathogens, but its effectiveness against *Shigella* species is inconsistent. A study analyzing non-*Salmonella* Gram-negative isolates from poultry found that all *Shigella sonnei* isolates were resistant to fosfomycin [14]. These findings indicate that fosfomycin's utility in treating *S. sonnei* infections, particularly *XDR* strains, may be limited and unpredictable.

Similarly, piperacillin, often combined with tazobactam to enhance its activity against beta-lactamase-producing organisms, has shown inconsistent efficacy. Data on its effectiveness against *XDR S. sonnei* are scarce, but studies have reported high resistance rates to piperacillin among *Salmonella* species, with resistance rates reaching up to 60.9% [15]. Given the genetic similarities between *Salmonella* and *Shigella*, it is plausible that *S. sonnei* may exhibit similar resistance patterns, potentially limiting piperacillin's role in treating XDR infections.

In contrast, meropenem remains a highly reliable option for managing *XDR S. sonnei* bacteremia due to its broad-spectrum activity and stability against beta-lactamases. Its effectiveness in treating multidrug-resistant Gram-negative infections has been well documented, and its use in this case led to rapid clinical improvement, further supporting its role as a preferred treatment.

In cases of *Shigella* bacteremia, testing for antibiotic susceptibilities is vital due to the increasing prevalence of antibiotic resistance. Preventive measures should focus on educating patients about safe practices postinfection to prevent transmission to close contacts.

## Data Availability

Data sharing is not applicable to this article as no new data were created or analyzed in this study.
